# Selective Precipitation of REE-Rich Aluminum Phosphate with Low Lithium Losses from Lithium Enriched Slag Leachate

**DOI:** 10.3390/ma17205113

**Published:** 2024-10-19

**Authors:** Vladimír Marcinov, Dušan Oráč, Jakub Klimko, Zita Takáčová, Jana Pirošková, Ondřej Jankovský

**Affiliations:** 1 Institute of Recycling Technologies, Faculty of Materials, Metallurgy and Recycling, Technical University of Košice, Letna 1/9, 04200 Košice-Sever, Slovakia; vladimir.marcinov@tuke.sk (V.M.); dusan.orac@tuke.sk (D.O.); jakub.klimko@tuke.sk (J.K.); zita.takacova@tuke.sk (Z.T.); jana.piroskova@tuke.sk (J.P.); 2 Department of Inorganic Chemistry, Faculty of Chemical Technology, University of Chemistry and Technology, 160 00 Prague, Czech Republic

**Keywords:** lithium-ion batteries, lithium-ion batteries recycling, lithium recycling, lithium slag, aluminum precipitation, solution refining, solution purification, hydrometallurgy, aluminum phosphate

## Abstract

Currently, recycling of spent lithium-ion batteries is carried out using mechanical, pyrometallurgical and hydrometallurgical methods and their combination. The aim of this article is to study a part of the pyro-hydrometallurgical processing of spent lithium-ion batteries which includes lithium slag hydrometallurgical treatment and refining of the obtained leachate. Leaching was realized via dry digestion, which is an effective method capable of transferring over 99% of the present metals, such as Li, Al, Co, Cu, and others, to the leachate. In this work, the influence of three types of precipitation agents (NaOH, NH_4_OH, Na_3_PO_4_) on the precipitation efficiency of Al and Li losses was investigated. It was found that the precipitation of aluminum with NaOH can result in the co-precipitation of lithium, causing total lithium losses up to 40%. As a suitable precipitating agent for complete Al removal from Li leachate with a minimal loss of lithium (less than 2%), crystalline Na_3_PO_4_ was determined under the following conditions: pH = 3, 400 rpm, 10 min, room temperature. Analysis confirmed that, in addition to aluminum, the precipitate also contains the REEs La (3.4%), Ce (2.5%), Y (1.3%), Nd (1%), and Pr (0.3%). The selective recovery of these elements will be the subject of further study.

## 1. Introduction

In recent years, the world has focused on environmental protection with the aim of achieving environmental and economic sustainability. Many organizations and countries are actively dedicating resources to efforts aimed at replacing traditional internal combustion engines with electric vehicles and devices powered by renewable energy sources. Recycling batteries and other energy storage components after the battery has exhausted its profitability for second life applications (e.g., stationary battery storage) is considered essential for the successful adoption of battery powered technologies. Recycling also provides an opportunity to reduce the life-cycle costs of batteries by recovering valuable materials and avoiding the expenses associated with the disposal of hazardous waste [1,2]. The utilization of new technologies necessitates a diverse range of materials, and European Union (EU) countries exhibit particularly high consumption levels. To meet this demand, EU nations not only rely on primary raw material extraction but also resort to importing raw materials, posing a significant risk to the supply chain and sustainable development. Mitigating the risk of material shortages can be achieved through the production of secondary raw materials, a primary objective of implementing a circular economy within key sectors of the EU [3].

Numerous studies have explored hydrometallurgical and pyrometallurgical approaches for the recycling of spent lithium-ion batteries (LiBs). Despite the diversity of metals present in spent lithium batteries such as lithium, nickel, cobalt, aluminum, iron, and others, hydrometallurgical methods have demonstrated effective recovery of these metals [4,5,6,7]. Nevertheless, processes involving the dismantling or disassembly of cells to obtain active cathode materials, as well as certain pyrometallurgical pre-treatment techniques, entail significant labor and power consumption. In contrast, industrialized methods like the Umicore VAL’EAS™ smelting technique offer a simpler alternative that does not necessitate mechanical pretreatment [7,8].

In the pyrometallurgical process, which does not necessitate the disassembly of spent LiBs, metals like Fe, Cu, Ni, and Co are reduced into an alloy. However, a drawback of all pyrometallurgical recycling methods for spent LIBs is that lithium tends to remain in the slag phase, aided by the incorporation of slag-forming agents like CaO and SiO_2_. Typically, the slags produced by pyrometallurgical processes are directly employed as a construction material or additive in cement manufacturing. But usage of lithium enriched slag as a construction material is not resource-efficient, particularly for lithium [8,9].

Cathode materials includes a range of elements compared to primary raw materials, including Co, Ni, Mn, Cu, Al, Fe, and others. While these metals can be recovered through both pyrometallurgical and hydrometallurgical processes, lithium can only be retrieved through hydrometallurgical treatment [10,11]. Lithium-containing slags are predominantly utilized in the production of cements, precursors for geopolymer production, and inorganic binders. However, this application may be considered as a misallocation of material potential [12,13,14].

There is only a limited number of studies that have focused on the utilization of lithium slags, and current research is focused on roasting and leaching of slags after the pyrometallurgical treatment of spent LiBs.

Dang et al. [15] utilized the addition of CaCl_2_ for chlorination roasting of lithium slags, followed by neutral leaching in water. Under optimal roasting conditions at 800 °C for 60 min with a molar ratio of Cl/Li = 1.8:1, a maximum lithium recovery of 90.58% was achieved. Subsequent leaching conditions at 60 °C for 30 min with a L:S ratio of 30 further contributed to successful lithium extraction. Li et al. [16] employed sulfate roasting of lithium slags. Optimal roasting conditions were experimentally determined at 800 °C for 60 min with a molar ratio of Na_2_SO_4_/Li = 3:1. This was followed by water leaching at 70 °C for 80 min using an L:S ratio of 30, resulting in a lithium recovery efficiency of 93.62%.

Dang et al. [17] also explored the roasting of lithium slags with the addition of K_2_CO_3_ and Na_2_CO_3_. Successful lithium extraction from the roasted slag was achieved through K_2_CO_3_/Na_2_CO_3_ roasting, followed by water leaching. Theoretical calculations suggested that Li–O bond lengths increase after the adsorption of K^+^/Na^+^, facilitating the easy release of Li^+^ from the LiAlSi_2_O_6_ lattice after roasting with K_2_CO_3_/Na_2_CO_3_. The extraction efficiency of lithium could reach 93.87% under optimal conditions at a roasting temperature of 740 °C, roasting time of 30 min, leaching temperature of 50 °C, leaching time of 40 min, and an L:S ratio of 10.

Xiao et al. [18] investigated the refining of products after pyrometallurgical processing of spent LIBs. Results indicated that 98.67% Cu, 99.84% Co, and 99.77% Ni were obtained by leaching alloy powders in 120 g·dm^–3^ sulfuric acid at 90 °C for 8 h with a L:S ratio of 100. Manganese and lithium were transferred to the slag, which was subsequently leached under the same conditions with a leaching efficiency of 44.30% for Mn and 50.28% for Li. The authors further proposed that utilizing sulfate roasting could enhance the efficiency of lithium and manganese leaching from slags.

Thermal treatment in the form of roasting with the addition of SO_4_^2−^, Cl^−^, or CO_3_^2−^ to slags that already have been subjected to the pyrometallurgical process is inappropriate from an economic and ecological point of view due to high energy consumption and greenhouse gases production. For this reason, it is necessary to transfer Li into solution by agitation leaching; however, during the leaching of aluminosilicates, silica gels are formed, which prevent filtration and reduce the efficiency of leaching by Li sorption. To prevent the formation of gels, a dry digestion method is employed, allowing for the subsequent recovery of lithium from solution. In a previous study, Klimko et al. [9] investigated the use of dry digestion (DD) for leaching slags after pyrometallurgical processing of spent LiBs, aiming to prevent the formation of H_4_SiO_4_. The input material consisted of slag from the reduction melting of pyrolyzed black mass with the addition of CuO and SiO_2_. The method involves mixing the slag with concentrated H_2_SO_4_, followed by adding H_2_O to the resulting mixture. The optimal component ratio for DD is 10 mL concentrated H_2_SO_4_ and 24 mL H_2_O per 10 g of slag. The mixtures obtained through DD were subsequently leached in 100 mL water with an efficiency of 92.12% for Li.

Refining the leach solution is a crucial step to avoid the co-extraction of impurities with lithium. During the refining process, there is a potential for lithium losses, which may reach up to 30%, depending on the initial concentration of lithium and the concentration of impurities in the solution [11].

The aim of this study is to compare methods for aluminum precipitation from the solution after Li slag dry digestion leaching and to prevent lithium losses during Al precipitation.

## 2. Materials and Methods

### 2.1. Analytical Methods

Chemical analysis of input materials and solution samples was performed by atomic absorption spectrometry (AAS) with a Varian SpectrAA20+ type spectrophotometer (Varian, detection limit: 0.3–6 ppb; slit width 0.2–1 nm; wavelength 213.9–422 nm; and lamp current 4–12 mA, Belrose, Australia). Elemental analysis was performed by using Energy-dispersive X-ray spectrometry (EDS) using an EDX-7000P spectrometer (Shimadzu, Kyoto, Japan). A thermodynamic analysis was performed using HSC 10 software (Outotec, Espoo, Finland). Analysis of precipitates was performed using a Panalytical Xpert Pro RV-11 (Philips, Amsterdam, The Netherlands) and SEM-EDS Mira3 Tescan (Brno, Czech Republic). Temperature and pH of the solution were measured by an inoLab, WTW 3710 (Xylem Analytics, San Diego, CA, USA). The particle size distribution was analyzed by a laser diffraction method (LD) using a Malvern Panalytical Mastersizer 3000 (Malvern, United Kingdom) device with a 4 mW He-Ne 632.8 nm Red light source and a 10 mW LED 470 nm Blue light source. The measurement was carried out in a wet cell using Isopropyl Alcohol (Penta, purity p.a. Prague, Czech Republic). During the measurement, the suspension was continuously mixed at a constant speed of 3000 rpm.

### 2.2. Leach Solution Preparation

**Figure 1:**
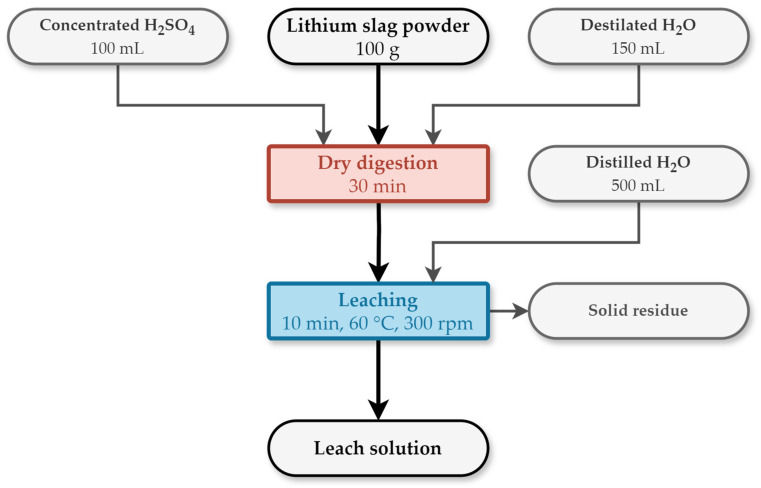
Schematic process flow sheet of leach solution preparation by Dry Digestion and leaching.

The solution was obtained by dry digestion leaching (Figure 1). The input material for DD leaching was electric arc furnace slag obtained by the pyrometallurgical treatment of pelletized black mass of spent LiBs with the addition of SiO_2_ and CuO [8]. The solution was prepared by leaching 100 g of Li slag with the addition of 100 mL of concentrated H_2_SO_4_ and 200 mL of deionized H_2_O. Subsequently, the material was leached in 500 mL of deionized H_2_O for 10 min at 60 °C and then filtered [9].

### 2.3. Precipiration Methodology

Precipitation was realized in glass beakers with a volume of 400 mL under constant stirring at 300 revolutions per minute (rpm), while the precipitating agent was added to the solution. Precipitation agents included: 2 M NaOH, NH_4_OH, a concentrated Na_3_PO_4_ solution, and crystalline Na_3_PO_4_. After reaching the required pH value (pH = 1 to pH = 12), the solution was stirred for 10 min in order to reach the equilibrium pH. Subsequently, the whole volume of solution was filtered, and a 10 mL sample was taken for chemical analysis. Precipitates were washed at 100 mL in deionized H_2_O at 20 °C for 10 min. A 10 mL sample was then taken for chemical analysis. SEM–EDX, XRD, and XRF analysis of the precipitates was performed after thermal treatment at 1100 °C for 60 min.

## 3. Results and Discussion

### 3.1. Leach Solution Preparation

Preparation of leach liquor involves mechanical pre-treatment of slag by size reduction and magnetic separation. Magnetic separation was used to increase the lithium concentration and to remove magnetic impurities, as shown in Table 1.

### 3.2. Input Leach Solution Analysis

Table 2 displays the results of the chemical analysis of the input leachate with an initial pH of 1.5. The highest concentration of metal ions in the solution is represented by aluminum (2666 mg/L) and lithium (2239 mg/L), followed by iron, copper, manganese, cobalt, and other metals. In terms of the value of metals in the leach solutions, lithium is the most valuable element for extraction, with a price of 381.4 $/m^3^ for Li_2_CO_3_. Other economically recoverable metals from the solution may include cobalt (13.9 $/m^3^), copper (7.8 $/m^3^), and aluminum (6.9 $/m^3^) as by-products of recycling (the listed prices related to 17 April 2024). However, elements such as nickel, manganese, and iron are considered impurities due to their low specific value.

The results of the AAS chemical analysis also confirmed that dry digestion effectively overcame the activation energy barrier for reactions, leading to the precipitation of SiO_2_ from the leach solution. The final concentration of silicon in the solution is only 1.4 mg/L [8].

The analysis confirmed that the leaching solution contains lithium, as the main marketable product, while the other present elements are considered as by-products or impurities. The direct recovery of lithium from the leach solution would result in a low-quality compound due to the high impurity content. Therefore, the removal of impurities from the solution has to be carried out. A theoretical study will be focused on the selective removal of aluminum with a focus on low lithium losses.

### 3.3. Thermodynamic Study of Metal Ions Precipitation

The first step of the study is a thermodynamic study, with the aim of identifying potential reactions of precipitation reagents (NaOH, NH_4_OH, Na_3_PO_4_) with metal ions in the leach solution.

**Figure 2:**
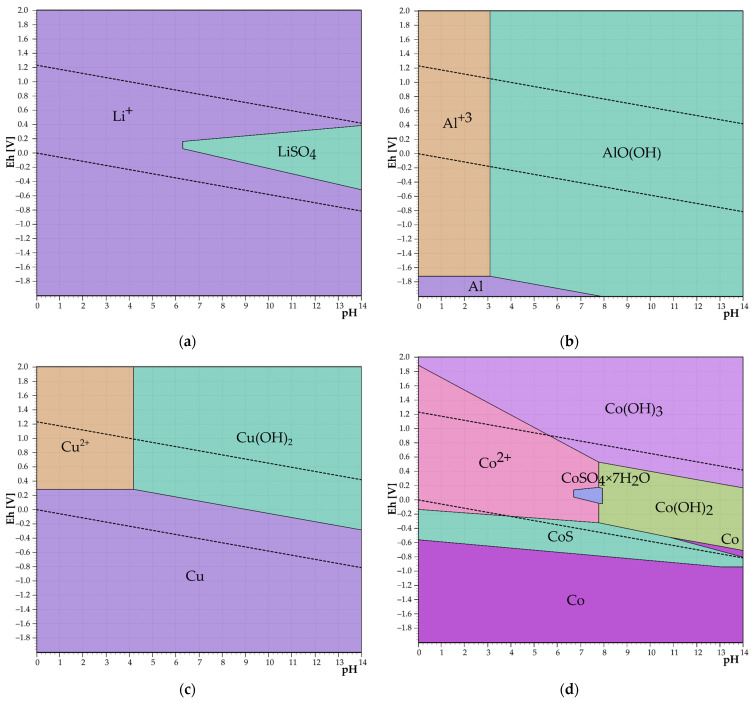

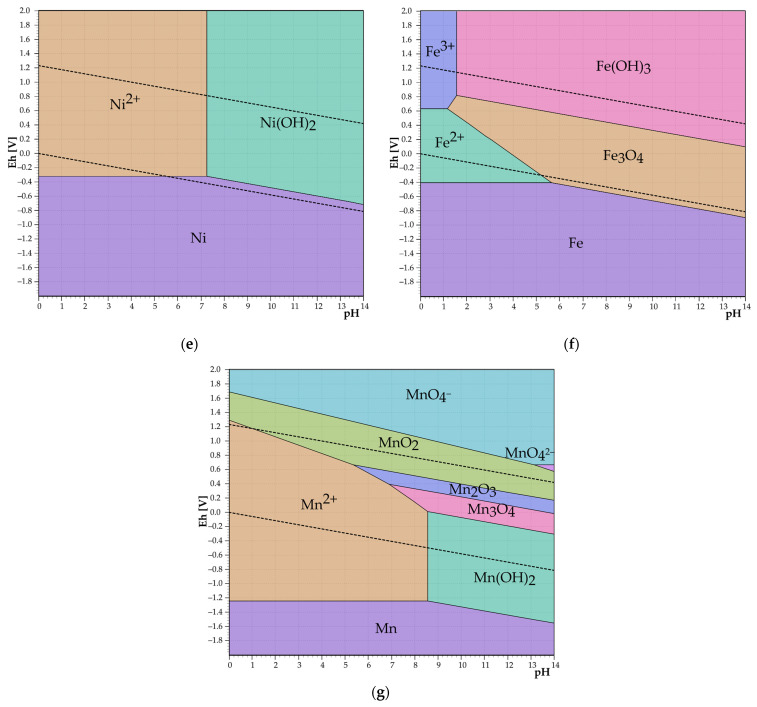
Eh–pH diagrams of precipitation of (**a**) Li, (**b**) Al, (**c**) Cu, (**d**) Co, (**e**) Ni, (**f**) Fe, (**g**) Mn in Me-S-H_2_O system at 20 °C.

Equations (1)–(8) show the possible course of reactions during precipitation with NaOH, together with ΔG°_293.15_ under laboratory conditions (20 °C, 1 atm). The temperature of 20 °C was chosen because of the higher solubility of Li salts, which should prevent Li losses during the precipitation of the present elements. Based on the negative values of free Gibbs energy, it is possible that Equations (1)–(8) will proceed. Eh–pH diagrams in Figure 2a,b determine that Li is not precipitated from the solution in the form of LiOH at the established pH interval and Al(OH)_3_ begins to precipitate from solution at pH = 3.7, and the precipitation should therefore proceed selectively. Figure 2c–g shows Eh–pH diagrams of precipitation of Cu, Co, Ni, Fe, and Mn. Cu starts to precipitate as Cu_2_O at pH = 3 and as Cu(OH)_2_ at pH = 4, and to coprecipitate at pH = 7.5 as Co(OH)_2_. The pH interval of NiSO_4_ · 4H_2_O is from 0.7 to 12.9 and Ni(OH)_2_ precipitates from pH 8 to 13.8.
Li_2_SO_4_ + 2NaOH = 2LiOH + Na_2_SO_4_ΔG°_293.15_ = −67.395 kJ(1)Al_2_(SO_4_)_3_ + 6NaOH = 2Al(OH)_3_ + 3Na_2_SO_4_ΔG°_293.15_ = −710.157 kJ(2)CuSO_4_ + 2NaOH = Cu(OH)_2_ + Na_2_SO_4_ΔG°_293.15_ = −221.123 kJ(3)CoSO_4_ + 2NaOH = Co(OH)_2_ + Na_2_SO_4_ΔG°_293.15_ = −186.431 kJ(4)NiSO_4_ + 2NaOH = Ni(OH)_2_ + Na_2_SO_4_ΔG°_293.15_ = −195.268 kJ(5)FeSO_4_ + 2NaOH = Fe(OH)_2_ + Na_2_SO_4_ΔG°_293.15_ = −177.835 kJ(6)Fe_2_(SO_4_)_3_ + 6NaOH = 2Fe(OH)_3_ + 3Na_2_SO_4_ΔG°_293.15_ = −723.961 kJ(7)MnSO_4_ + 2NaOH = Mn(OH)_2_ + Na_2_SO_4_ΔG°_293.15_ = −68.596 kJ(8)

NH_4_OH was chosen as another precipitating agent to avoid contamination of the solution with sodium. Equations (9)–(16) show the possible course of reactions during precipitation with NH_4_OH, together with ΔG°_293.15_ under laboratory conditions (20 °C, 1 atm). Compared to NaOH as a precipitating agent, the ΔG°_293.15_ values are higher against NaOH. However, ΔG°_293.15_ is still negative, which means that the equilibrium of the reaction will be on the side of products. When precipitation takes place in an OH^−^ environment, the Eh–pH diagrams, and thus it is possible to conclude that precipitation of metal ions considered as impurities, will take place selectively from lithium.
Li_2_SO_4_ + 2NH_4_OH = 2LiOH + (NH_4_)_2_SO_4_ΔG°_293.15_ = −20.615 kJ(9)Al_2_(SO_4_)_3(ia)_ + 6NH_4_OH = 2Al(OH)_3_ + 3(NH_4_)_2_SO_4_ΔG°_293.15_ = −251.166 kJ(10)CuSO_4(ia)_ + 2NH_4_OH = Cu(OH)_2_ + (NH_4_)_2_SO_4_ΔG°_293.15_ = −86.801 kJ(11)CoSO_4(ia)_ + 2NH_4_OH = Co(OH)_2_ + (NH_4_)_2_SO_4_ΔG°_293.15_ = −52.173 kJ(12)NiSO_4(ia)_ + 2NH_4_OH = Ni(OH)_2_ + (NH_4_)_2_SO_4_ΔG°_293.15_ = −50.093 kJ(13)FeSO_4(ia)_ + 2NH_4_OH = Fe(OH)_2_ + (NH_4_)_2_SO_4_ΔG°_293.15_ = −61.720 kJ(14)Fe_2_(SO_4_)_3(ia)_ + 6NH_4_OH = 2Fe(OH)_3_ + 3(NH_4_)_2_SO_4_ΔG°_293.15_ = −346.692 kJ(15)MnSO_4(ia)_ + 2NH_4_OH = Mn(OH)_2_ + (NH_4_)_2_SO_4_ΔG°_293.15_ = −32.771 kJ(16)

**Figure 3:**
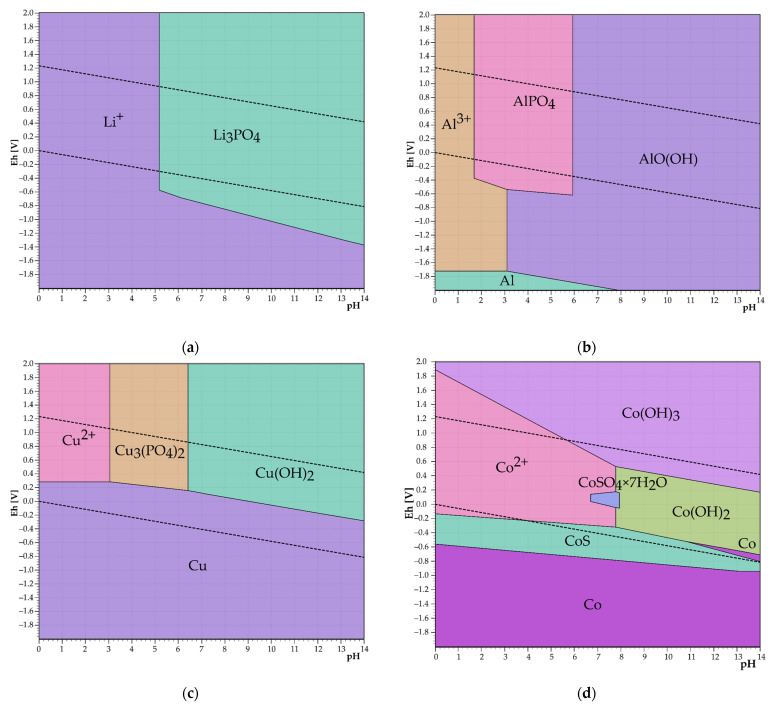

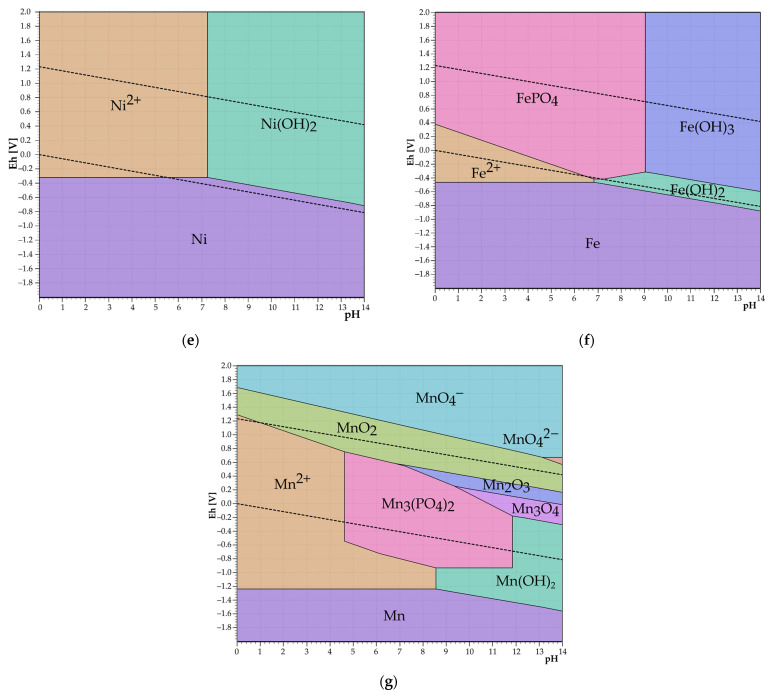
Eh–pH diagrams of precipitation of (**a**) Li, (**b**) Al, (**c**) Cu, (**d**) Co, (**e**) Ni, (**f**) Fe, and (**g**) Mn in Me–P–S–H_2_O system at 20 °C.

Equations (17)–(24) show the reactions of sulfates present in the leachate with a Na_3_PO_4_ precipitating reagent. The formation of Co, Ni, and Fe^2+^ precipitates in the PO_4_^3−^ environment all have positive ΔG° values, which means that these precipitates should not form. From the Eh–pH diagrams in Figure 3a,b, it is clear that Al in the form of AlPO_4_ precipitates in the leachate at pH = 1–5 and subsequently, an AlO(OH) precipitate is formed. Lithium starts to precipitate in the form of phosphate at pH = 5 thus precipitation could be considered as selective in case of using PO_4_^3−^ as a precipitating agent for aluminum. Copper should form a precipitate right after the addition of PO_4_^3−^, and iron should form a FePO_4_ · 2H_2_O precipitate at pH = 0–5.5.
1.5Li_2_SO_4(ia)_ + Na_3_PO_4(ia)_ = Li_3_PO_4_ + 1.5Na_2_SO_4(ia)_ΔG°_293.15_ = −68.570 kJ(17)Al_2_(SO_4_)_3(ia)_ + 2Na_3_PO_4(ia)_ = 2AlPO_4_ + 3Na_2_SO_4(ia)_ΔG°_293.15_ = −225.567 kJ(18)3CuSO_4(ia)_ + 2Na_3_PO_4(ia)_ = Cu_3_(PO_4_)_2_ + 3Na_2_SO_4(ia)_ΔG°_293.15_ = −50.039 kJ(19)3CoSO_4(ia)_ + 2Na_3_PO_4(ia)_ = Co_3_(PO_4_)_2_ + 3Na_2_SO_4(ia)_ΔG°_293.15_ = 9.509 kJ(20)3NiSO_4(ia)_ + 2Na_3_PO_4(ia)_ = Ni_3_(PO_4_)_2_ + 3Na_2_SO_4(ia)_ΔG°_293.15_ = 6.207 kJ(21)3FeSO_4(ia)_ + 2Na_3_PO_4(ia)_ = Fe_3_(PO_4_)_2_ + 3Na_2_SO_4(ia)_ΔG°_293.15_ = 6.461 kJ(22)Fe_2_(SO_4_)_3(ia)_ + 2Na_3_PO_4(ia)_ = 2FePO_4_ + 3Na_2_SO_4(ia)_ΔG°_293.15_ = −74.648 kJ(23)3MnSO_4(ia)_ + 2Na_3_PO_4(ia)_ = Mn_3_(PO_4_)_2_ + 3Na_2_SO_4(ia)_ΔG°_293.15_ = −176.004 kJ(24)

The thermodynamic study showed that precipitation of aluminum and other impurities from lithium enriched slag after dry digestion should be selective in case of hydroxide precipitation. Removal of aluminum by phosphate precipitation should be selective at pH = 0–5. After reaching pH = 5, lithium phosphate is precipitated along with other impurities such as copper, nickel, and manganese.

### 3.4. Precipitation Results and Discussion

**Figure 4:**
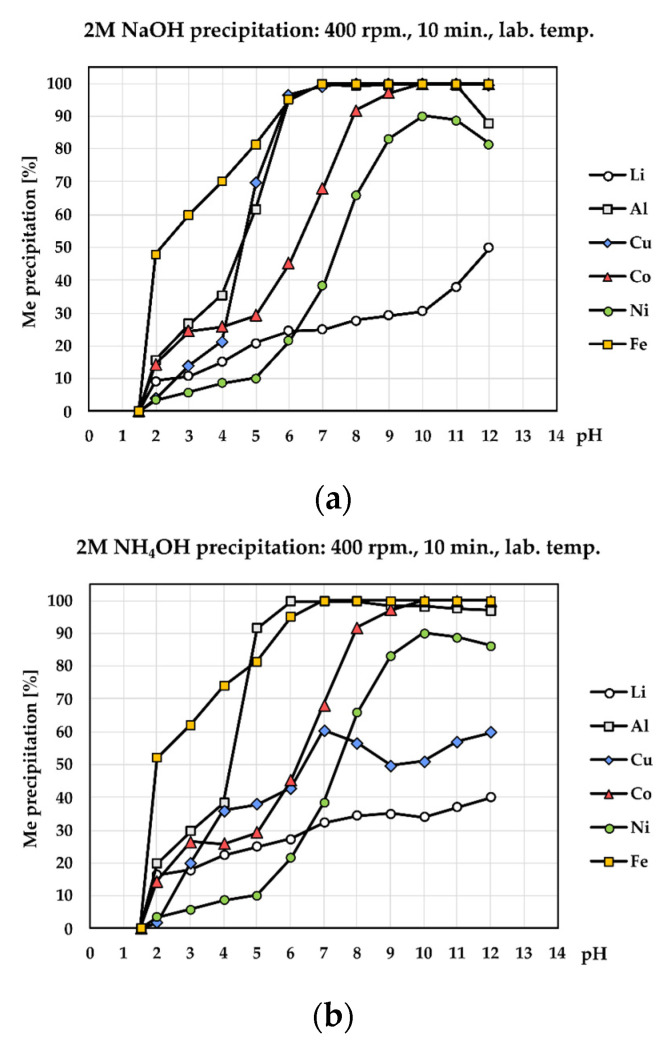
Precipitation efficiency of present metals including Li losses (**a**) using 2 M NaOH, (**b**) using 2 M NH_4_OH.

**Figure 5:**
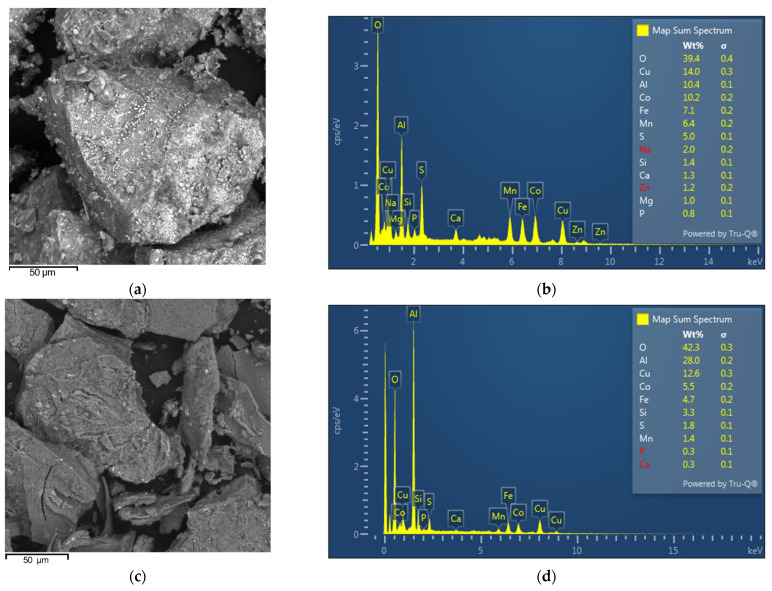
(**a**) SEM of precipitate with NaOH, (**b**) corresponding EDS spectrum, (**c**) SEM of precipitate with NH_4_OH, (**d**) corresponding EDS spectrum.

Figure 4 shows the precipitation process using 2 M NaOH (Figure 4a) and 2 M NH_4_OH (Figure 4b). As can be seen, aluminum started to precipitate immediately after the addition of the NaOH solution and was completely removed from the solution at pH = 7 using both reagents. The concentration of Al started to rise from pH = 8.5. Significant losses of Li occur during precipitation of Al. At pH = 7, the concentration of Li decreased by 25.03% (2 M NaOH). Similar losses of Li can also be observed using NH_4_OH. Complete removal of Al occurred at pH = 6 and lithium losses were 28%. Precipitation is nonselective due to relatively high concentrations of Cu, Co, Fe, and Mn, which confirms the SEM–EDS analysis of selected precipitates shown in Figure 5.

Lithium losses are probably caused by the formation of lithium aluminum oxide hydrate. The formation of LiAl_5_O_8_ is confirmed by R. Ribeiro et al. [19] who prepared LiAl_5_O_8_ by precipitation from a solution of Al(NO_3_)_3_ · 9H_2_O and LiNO_3_ by adding NaOH in a ratio of 1:1.2:4.

T.R.N. Kutty and M. Nayak [20] synthesized LiAl_5_O_8_ from an aqueous LiOH solution by adding KAl(SO_4_)_2_ · 12H_2_O as a source of Al and NaOH to adjust the pH of the solution. From which it follows that the formation of LiAl_5_O_8_ from an aqueous solution is possible, which can explain the high losses of Li during the precipitation of Al through hydroxides. The formation of lithium–aluminum hydroxides confirms research by Shuaike LV et. al. [21]. Lithium aluminum layered double hydroxides (LiAl-LDHs) have emerged as the most promising adsorbent for lithium extraction from salt lake brines. LiAl-LDHs are fabricated by intercalating lithium ions, typically sourced from compounds like LiCl, LiOH, or (Li_2_SO_4_), into aluminum hydroxides. These aluminum hydroxides are often found in the form of naturally occurring minerals such as gibbsite or bayerite. The techniques for producing LiAl-LDHs include solid-state synthesis, coprecipitation, and hydrothermal methodology [22]. High lithium losses can be explained by the formation of LiAl-LDHs or sorption of lithium on the surface of Al(OH)_3_.

**Figure 6:**
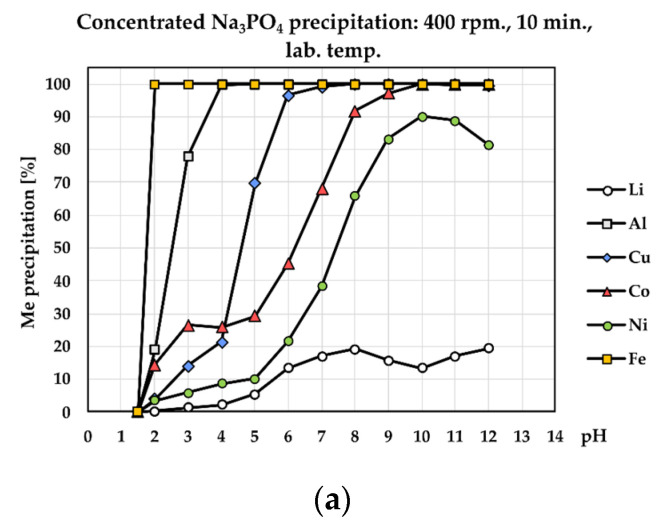

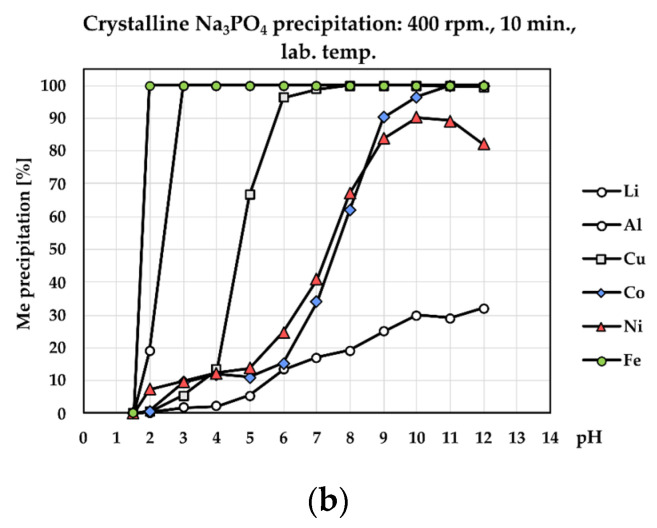
Precipitation efficiency of present metals including lithium losses (**a**) using concentrated Na_3_PO_4_ solution and (**b**) using crystalline Na_3_PO_4_.

SEM-EDS analysis of a precipitate with NaOH (Figure 5a) showed similar surface morphology with a NH_4_OH precipitate (Figure 5c); however, precipitation with NaOH produced a bigger particle size of precipitate. EDX spectra of both precipitates (Figure 5b,d) showed that precipitation of aluminum using NaOH and NH_4_OH is non-selective and during precipitation high lithium losses were observed. The second method relates to the precipitation of Al using a concentrated solution of Na_3_PO_4_ and crystalline Na_3_PO_4_. As can be seen (Figure 6a), the complete removal of Al from the solution occurred using crystalline Na_3_PO_4_ at pH = 3. Lithium losses represented only 1.73%. Using a concentrated solution of Na_3_PO_4_, aluminum was removed at pH = 4 and lithium losses were 2.25%. A similar result was achieved by Chernyaev at. al. [23], which demonstrates that the use of phosphoric acid in phosphate precipitation was found to be more efficient than the more conventional solution purification by hydroxide precipitation. Phosphate precipitation was characterized by a rapid reaction between the phosphate and trivalent metal ions, with less than 2% of the valuable battery metals (Li, Co, Ni) incorporated in the phosphate precipitate.

**Figure 7:**
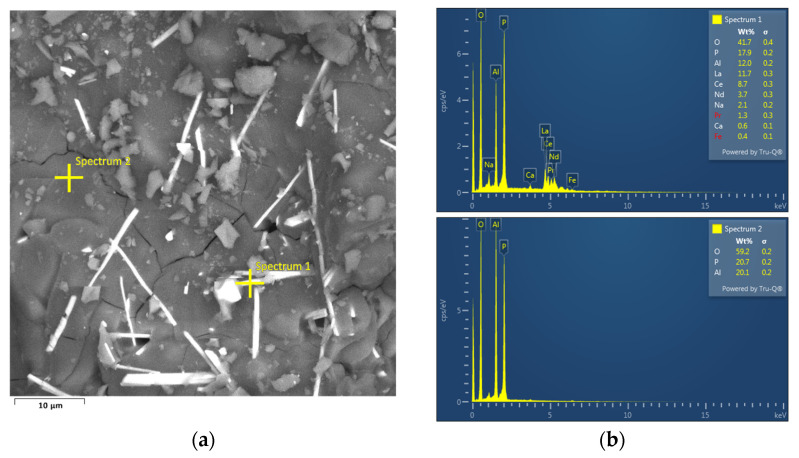
(**a**) SEM of precipitate by crystalline Na_3_PO_4_ and (**b**) point EDS spectrum.

The lowest lithium losses were achieved at pH = 3 by precipitation of aluminum with crystalline Na_3_PO_4_. The lower pH and higher precipitation efficiency can be explained by local supersaturation of the solution by PO_4_^3−^ near the particle surface, and by the solid phase in solution that serves as a nucleation core [24]. Precipitates formed by crystalline Na_3_PO_4_ are covered by needle-shaped crystals (Figure 7a). One of the EDS spectra (Figure 7b) shows the presence of rare earth elements (REEs) on a surface of the needle crystals at the point labeled “Spectrum 1”.

**Figure 8:**
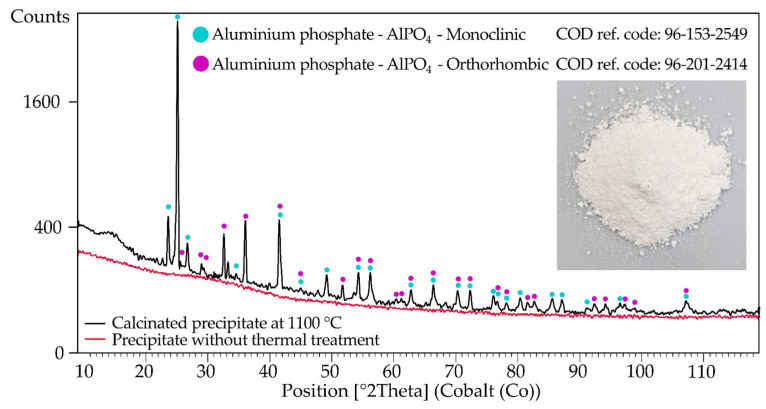
XRD pattern of aluminum phosphate precipitate obtained by crystalline Na_3_PO_4_ (red) and calcinated precipitate at 1100 °C shifted up by 50 counts (black).

The SEM-EDX analysis confirms a high level of selectivity of aluminum precipitation from solution. XRD (Figure 8) and XRF analysis (Table 3) of the precipitate proves the formation of aluminum phosphate from solution with a low level of impurities, such as Co, Cu, Ni, and Mn. The XRF analysis confirmed a high content of REEs (Y, La, Ce, Nd, Pr).

**Figure 9:**
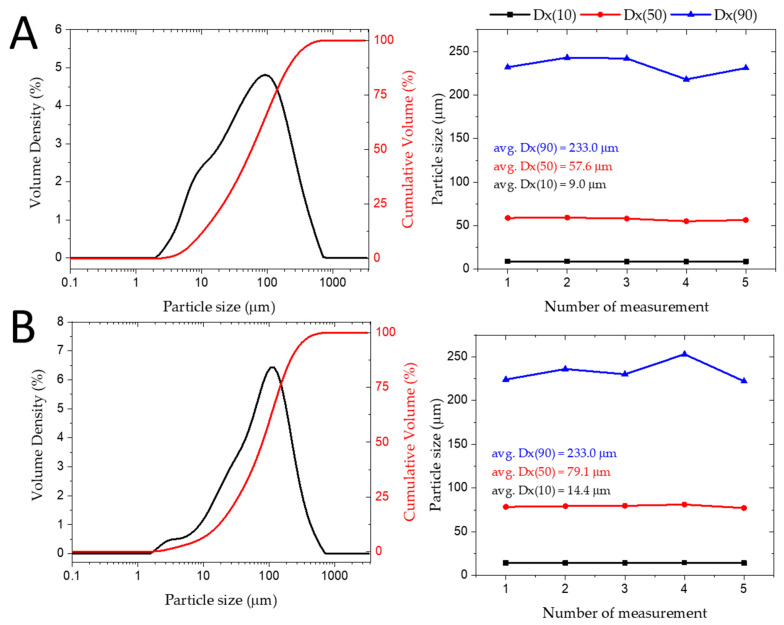
Particle size distribution of precipitated AlPO_4_ phase, (**A**) precipitate before calcination (**B**) calcinated precipitate at 1100 °C.

The presence of rare earth elements can be explained as follows: REEs are used as dopants to achieve better electrochemical properties of batteries in older technologies such as Ni–Cd and Ni–MH, as well as in lithium-ion batteries. Lithium-ion cathode materials doped with rare earth elements (La, Nd, Pr, Ce, Y, Eu) of various concentrations have much better rate performance, higher capacity retention, and higher Li+ diffusion coefficients than the pristine cathode materials. This fact can explain the relatively high content of REEs (as shown in Table 3) in the phosphate precipitate after calcination at 1100 °C from the lithium slag leachate and Figure 9 shows particle size distribution, which confirms that 90% of particles are under 233 µm [25,26,27].

**Figure 10:**
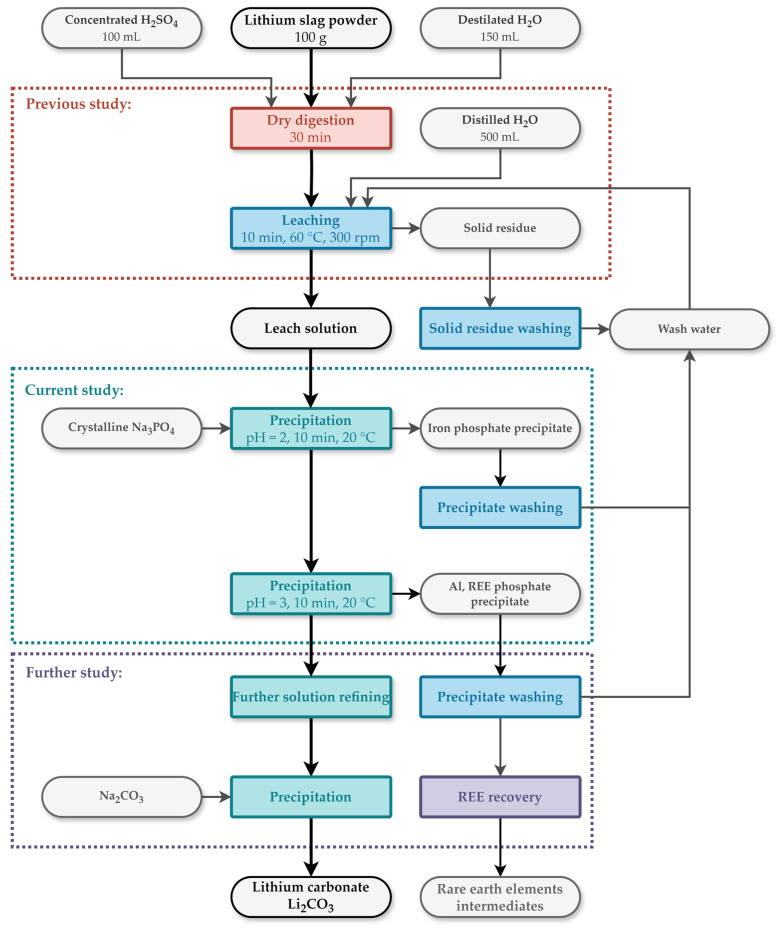
Flowsheet of lithium enriched slag treatment by dry digestion, leaching and precipitation.

Figure 10 shows a flow sheet of the current state of the lithium slag recycling process. The first step of the flowsheet is a dry digestion leaching of slag by mixing it with concentrated H_2_SO_4_ and water. Subsequently, refining of the leached solution is required. The biggest lithium losses were observed following aluminum precipitation by hydroxides [11,28]. Therefore, it was necessary to prevent lithium losses. For these reasons, the proposal includes Na_3_PO_4_ as a suitable precipitating agent at pH = 3 at 20 °C for effective and selective aluminum precipitation. In this case, lithium losses reach only 1.73%. However, it is possible to selectively precipitate FePO_4_ at pH = 3 at 20 °C. Further REE recovery can be done by industrialized processes for REE recovery, which will be topic for further study.

A summary of the results of the precipitation experiments, including the precipitation efficiency, the metal content in leachate after precipitation, and the loss of metals of interest, particularly lithium, are given in Table 4.

## 4. Conclusions

In the pyrometallurgical process of spent LiBs recycling, metals such as Fe, Cu, Ni, and Co are reduced into an alloy. The main advantage of pyrometallurgical treatment is the availability of large-scale processing of various battery chemistries (e.g., NMC, LCA, LCO, LFP etc.). However, a limitation of all pyrometallurgical recycling methods for spent LIBs is that lithium tends to remain in the slag phase, thus it is important to focus on recovery of lithium from pyrometallurgical slags containing lithium. In order to fully recover the material potential of spent LiBs, it is necessary to implement combined pyrometallurgical and hydrometallurgical processes. For the extraction of metals from lithium enriched slag, the following aspects are associated with hydrometallurgical processing:

- Dry digestion is a highly efficient way of leaching lithium enriched slags with a leaching efficiency of lithium of up to 99% and the prevention of silica gel formation during leaching.
- Direct and selective recovery of pure lithium marketable products (Li_2_CO_3_/LiOH) from sulfate solutions is not possible, therefore solution refining is necessary. The main challenge of solution refining is lithium loss during aluminum removal, which can reach up to 40% by conventional hydroxide precipitation methods and losses cannot be reduced by water washing.
- Precipitation of aluminum by OH^−^ is not suitable, due to high lithium losses (30%).
- Precipitation of aluminum by crystalline Na_3_PO_4_ is highly selective with minimal lithium losses equal to 1.7% after washing of the precipitate, lithium losses can be minimized to 0.2% and wash water can be used as an input for dry digestion leaching. Losses of Co (19.8%) and Cu (5.4%), as secondary possible marketable products, can be minimized to Co (1.7%) and Cu (1.3%) by water washing of the precipitate.
- By using phosphate, the selective recovery of iron is possible at pH = 2.
- Losses of lithium, cobalt, and copper during phosphate precipitation of aluminum are caused by physical sorption and losses can be minimized by water washing of the precipitates.
- The SEM-EDX and XRF analyses revealed a high content of the rare earth elements La (3.447%), Ce (2.542%), Y (1.349%), Nd (1.01%), and Pr (0.318%) concentrated in the phosphate precipitate.

The proposed recycling process with the use of phosphate precipitation, allows minimization of lithium losses, and compared to other combined methods, processes of slag treatment using an external heat source are not required due to the fact that dry digestion is an exothermic process and high lithium leaching efficiencies are achieved at ambient laboratory temperatures. Additionally, the implementation of phosphate precipitation in existing recycling facilities does not require significant changes in the equipment used. Further research should focus on REE recovery from aluminum phosphate precipitate and solution refining with the aim of eliminating lithium losses and achieving high-yield lithium recovery.

Developing technologies for the valorization of secondary resources can lead to a reduction of primary material mining and import dependency. Recycling of spent LiBs enables the recovery of compounds, which can then be reused in the production industry. This approach contributes to a more sustainable and circular economy.

## Figures and Tables

**Table 1:** Chemical composition of lithium slag before and after magnetic separation.

Analyte	Li	Co	Al	Cu	Ni	Fe	Mn	Si
Lithium slag ^1^ [%]	2.96	1.69	10.3	2.58	0.11	1.07	0.94	36.65
Lithium slag ^2^ [%]	3.68	1.17	11.02	1.71	0.11	1.26	0.97	30.29
Magnetic fraction [%]	0.16	28.94	0.26	46.59	3.22	11.28	0.56	14.8

^1^ Chemical composition of slag before magnetic separation. ^2^ Chemical composition of slag after magnetic separation.

**Table 2:** The chemical analysis of the input leach solution and specific material value of individual metal ions in solution (AAS).

Analyte	Li	Co	Cu	Al	Ni	Fe	Mn	Si
Me concentration [mg/L]	2239.24	499.05	827.46	2666.96	36.05	930.4	514.1	1.4
Me molarity [mM]	322.72	8.46	13.02	98.85	0.61	16.65	9.36	0.05
Value of Me in solution [$/m^3^]	381.41 *	13.889	7.77	6.85	0.64	0.10	0.01	-

* Marketable product of Li is Li_2_CO_3_ containing 18.787% of lithium

**Table 3:** The XRF analysis of aluminum phosphate precipitate obtained by crystalline Na_3_PO_4_ after calcination at 1100 °C.

**Element**	**P**	**Al**	**Na**	**Si**	**S**	**Mn**	**Fe**	**Co**
Wt. %	50.29	29.72	2.58	1.45	0.02	0.18	3.20	0.10
**Element**	**Ni**	**Cu**	**Y**	**Zr**	**La**	**Ce**	**Nd**	**Pr**
Wt. %	0.09	1.13	1.35	0.20	3.48	2.54	1.00	0.32

**Table 4:** Concentration of metal ions in solution after Na_3_PO_4_ precipitation.

Me Concentration	Li	Co	Al	Cu	Ni	Fe	Mn	Si
Lithium slag [%]	3.68	1.17	11.02	1.71	0.11	1.26	0.97	30.29
Leach solution [mg/L]	2239	499	2666	827	36.1	930	514	1.4
After precipitation	2200	400	0	782	31.6	0	501	0
Me removal [%]	1.7	19.8	100	5.5	12.3	100	2.3	100
Me losses * [%]	0.2	1.7	100	1.3	12	100	0.6	100

* Water washing of precipitate at 20 °C, 100 mL, 10 min.

## Data Availability

According to open science principles, raw data from laser diffraction can be found at DOI: https://zenodo.org/records/11160813 (accessed on 9 May 2024).
